# Phone triage nurses’ assessment of respiratory tract infections – the tightrope walk between gatekeeping and service providing. A qualitative study

**DOI:** 10.1080/02813432.2021.1908715

**Published:** 2021-04-01

**Authors:** Bent Håkan Lindberg, Ingrid Keilegavlen Rebnord, Sigurd Høye

**Affiliations:** a Antibiotic Centre for Primary Care, Department of General Practice, Institute of Health and Society, University of Oslo, Oslo, Norway; b National Centre for Emergency Primary Health Care, NORCE Norwegian Research Centre, Bergen, Norway; c Department of Global Public Health and Primary Care, University of Bergen, Bergen, Norway

**Keywords:** Out-of-hours, primary health care, phone triage, respiratory tract infections, nurses’ role, qualitative research

## Abstract

**Background:**

Phone nurses triage callers to Norwegian out-of-hours cooperatives to estimate the appropriate urgency and level of care for the caller. Many callers with mild symptoms of respiratory tract infections receive a doctor’s consultation, which may lead to busy sessions and in turn impair clinical decisions.

**Objective:**

This study explores how phone triage nurses assess callers with mild-to-moderate symptoms of respiratory tract infections and their views and experiences on triaging and counselling these callers.

**Methods:**

We conducted four focus groups with 22 nurses (five men and 17 women aged 24–66 years) in three different locations in Norway. The interviews were transcribed verbatim and analysed by systematic text condensation.

**Results:**

The informants were reluctant to call themselves gatekeepers. However, their description of their work indicates that they practice such a role. When nurses and callers disagreed about the right level of care, the informants sought consensus through strategies and negotiations. The informants described external factors such as organisational or financial issues as decisive for the population’s use of out-of-hours services. They also described callers’ characteristics, such as language deficiency and poor ability to describe symptoms, as determining their own clinical assessments.

**Conclusions:**

Nurses perceive assessments of callers with respiratory tract infections as challenging. They need skills and time to reach a consensus with the callers and guide them to the right level of health care. This should be considered when planning nurse training and staffing of out-of-hours cooperatives.

**KEY-POINTS**
- Phone triage nurses assess callers to the out-of-hours service and estimate the level of urgency
- This study explores how phone triage nurses assess callers with respiratory tract infections and their views and experiences on this task
- The nurses describe their professional role as a tightrope walk between gatekeeping and service providing
- The nurses seek consensus with callers through strategies and negotiations

## Introduction

In many middle- and high-income countries, out-of-hours primary care is provided by large-scale General Practitioners (GP) cooperatives staffed by nurses and doctors [1,2].

In Norway, GPs work shifts in the municipality-owned out-of-hours cooperatives and serve as gatekeepers to secondary health care. The population is encouraged to call, rather than showing up when they perceive that they need medical attention out of hours. Nurses assess these phone calls and decide whom they can handle by phone counselling alone, who can wait home and contact the regular GP the next day, and who needs an appointment with a doctor in the out-of-hours GP cooperative. Hence, the number of calls, and in turn how many callers the nurses assign for a doctor’s appointment, determine how busy each session will be for the on-duty doctors.

There is concern about overuse of out-of-hours health-care services [3]. Too many people seeking help from a service with limited resources will lead to busy sessions and inevitably allow less time to take care of patients in the highest need of care. Busy sessions may also impair the quality of care, as demonstrated in the case of inappropriate antibiotic prescribing [4]. In addition, a low threshold for professional evaluation of mild infections might reduce the population’s belief in self-evaluation over time [5].

In Norwegian out-of-hours service, there were 252 consultations per 1000 inhabitants in 2019 [6]. In 2014, nurses managed 23% of all incoming calls by phone counselling alone [7]. Respiratory tract infections are mostly self-limiting and a majority of people with these infections do not need to see a doctor for strict medical reasons. However, respiratory tract infections constitute almost 16% of all consultations in out-of-hours health care in Norway [6]. Most studies on the performance of nurse triage focus on acute, life-threatening conditions and show that it works well for high urgency cases [8]. To our knowledge, no studies have focused on the assessment of callers with mild-to-moderate conditions, such as respiratory tract infections, on how nurses judge their own role in these assessments, nor on nurses’ own conception of the gatekeeping role for these callers. Understanding why so many people with mainly self-limiting conditions prefer to seek medical care after hours and why the gate is held open for them may contribute to more rational use of out-of-hours primary health-care services.

This study aimed to explore how nurses assess callers with mild-to-moderate symptoms of respiratory tract infections and their views and experiences on triaging and counselling these callers.

BHL is a GP. He has worked shifts in an out-of-hours cooperative for almost 20 years and been the head physician in the same cooperative for the last 6 years. IKR is a long time GP and a researcher. She is a former head physician in an out-of-hours cooperative. SH is a GP and a long-time researcher in the field of antibiotic prescribing and respiratory tract infections. We share a common interest in factors influencing clinical assessments in general, and antibiotic prescribing in primary care in particular.

## Design, materials and methods

This qualitative study is based on four focus group interviews. Two groups were interviewed in their own out-of-hours GP cooperatives in two different counties in June and September 2018. The groups comprised only the nurses working at the particular cooperative. The other two group interviews were held during the annual conference for the primary care out-of-hours service in Norway [Den nasjonale legevaktkonferansen 2018] in September 2018. The two first groups were recruited through direct contact with their leaders by e-mail and telephone. For the last two groups, we did a purposive sample recruitment from the conference's participatory list. We contacted potential candidates by e-mail, aiming for variety in gender, age, size of out-of-hours cooperative and geographical location. We continued the recruitment process until we reached the desired number of six participants in each group.

The 22 participants’ (five men, 17 women) median age was 42 years (24–66 years). Their median out-of-hours service experience was 7.5 years (1–22 years). The participants represented out-of-hours GP cooperatives from all Norwegian regions. One of the groups consisted of women only. Table 1 presents the characteristics of the participants and the out-of-hours GP cooperatives.

We developed an interview guide that covered four main themes: (1) General considerations about respiratory tract infections in the out-of-hours service. (2) Reasons why persons with mild symptoms of respiratory tract infections receive appointments to see a doctor after hours. (3) Experience with nurse counselling solely of callers with respiratory tract infections. (4) Factors promoting or hindering a strategy to make the callers wait to see their regular GP the next day. The interviews lasted around 90 min and were recorded digitally. Two of the authors (BHL and SH) acted as moderators. The interview guide was followed pragmatically and adjusted after each focus group. Ideas and experiences that emerged and were discussed in early focus group interviews, e.g. the term gatekeeper, were introduced in later focus group interviews.

### Analysis

BHL transcribed all the interviews. BHL and SH analysed the transcripts following systematic text condensation using a four-step method [9]: (1) We read the whole text to obtain total impressions and identify preliminary themes. (2) We Identified meaning units and established code groups concerning the process of assigning doctor’s appointment to callers with symptoms of respiratory tract infections and sorted the meaning units correspondingly. (3) We systematically abstracted condensates and summarized the contents from each code group. (4) Finally, we generalized the descriptions and concepts for each theme.

IKR read the synthesised descriptions and then the transcripts, thus controlling the interpretations and results backwards.

BHL translated the informants’ quotes into English and gave them fictitious names.

NVivo software was used to manage the data [10].

The informants gave their informed consent to participate in the study and the Norwegian Centre for Research Data approved data protection (58953/3/EPA).

## Results

Four result categories emerged from the transcript. The informants described their professional practise as a tightrope walk between gatekeeping and service providing. They sought consensus with the callers, using strategies and negotiations, and they described both structural factors and callers’ characteristics influencing their assessments and the use of the out-of-hours service.

### Nurses’ professional practise – a tightrope walk between gatekeeping and service providing

The nurses answered phone calls from the public and managed the doctors’ schedule in all out-of-hours GP cooperatives that our informants represented. They described a reality where the doctor’s accessibility had to be restricted for capacity reasons; hence, they described a de facto gatekeeping role. However, several of our informants spontaneously expressed that they did not regard themselves as gatekeepers for the out-of-hours service.

In spite of these statements, the general attitude among the informants was that the out-of-hours service existed for what they judged as urgent medical matters. They expressed that whatever could wait, should wait. The informants wanted to assign doctor’s appointments primarily based on their medical judgement.

Oliver: It irritates me when people call and say that they want to reserve an appointment. That makes me quite rigid. These people really have to be sick to get an appointment. “Reserving an appointment” is not how things work here. The out-of-hours service is for those who really need it.

At the same time, the informants indicated that ‘service provider’ was the best description of their conception of their own role. To provide service was not necessarily to give in to the caller’s demands, but to provide what they considered was the best level of care for the caller. If the nurses judged that the best level of care was a next-day appointment with the callers’ GP, the provided service was to deny the request for an appointment in the out-of-hours GP cooperative. In this way, they were able to overcome an apparent antagonism between gatekeeping and service-providing roles.

The informants explained that it could be difficult to distinguish between the callers’ demands and needs. All described recurrent experience with callers who wanted to see a doctor in the out-of-hours GP cooperative, where the nurses judged it unnecessary from a medical point of view. In these situations, the most common strategy was to seek consensus by convincing the caller that it was not necessary to see a doctor out-of-hours for these particular symptoms. When consensus was impossible to reach, the solution was usually to grant the caller the desired appointment.

Mathilda: In a way, it depends on how angry they get. I very seldom quarrel. If we must argue our way through the call, I would rather give them a doctor’s appointment.

Some of the informants described a constant uncertainty concerning their own assessment of callers. This feeling of uncertainty could remain for hours after their shift ended. They feared having missed symptoms indicating grave disease or they feared an unexpected and serious infection. Their previous experiences with the unpredictable and abrupt progress of disease contributed to this fear. Hence, the informants considered it a huge responsibility not to give callers a doctor’s appointment. Several talked about the necessity for detailed documentation of their assessment and the advice given. The informants assumed that this would support the nurse’s decision in the case of an unexpected and serious progress of respiratory tract infections and an eventual injury complaint.

Megan: You never sit in that chair and feel confident.

The nurses considered triage scales as useful tools to assess callers with high-emergency conditions. For callers with respiratory tract infections, however, they considered triage scales inferior to their own clinical assessments, because this group normally would be considered low urgency on triage scales. Hence, as the triage scales gave no decision support for these callers, a clinical judgment of high urgency would always overrule the possible low urgency as indicated by a triage scale.

The informants discussed how they assessed particular doctors and their antibiotic prescribing patterns. Opinions differed as to whether keeping patients away from high prescribers, was a task for nurses. For some, being on duty with a low prescriber worked as an argument to keep callers away from the out-of-hours cooperative. They would tell the caller that there was no need to come to a consultation because the doctor would not prescribe antibiotics.

### Strategies and negotiations towards consensus

The informants described situations where a caller wanted to see a doctor in the out-of-hours GP cooperative and the nurse judged it unnecessary due to low level of urgency (green code) as situations similar to negotiations. In these negotiations, different strategies were used to achieve consensus that it would be safe for the caller to not have a doctor’s appointment out-of-hours. One of the strategies they described in the reassuring process was the triage itself. Several informants talked about how a low level of urgency and its corresponding green colour code, as opposed to medium (yellow code) and high (red code) level of urgency, became an argument to stay home or seek the GP later. This could also be an argument of convenience: i.e. a green code would mean a long wait time at the out-of-hours GP cooperative; therefore, it would be better for the caller to observe the situation at home.

Ruby: Being green is good for you, actually.

Another strategy described by the informants was to appear confident and convincing. The informants discussed how this communication technique could make it easier to come to an agreement with the caller about other measures than seeing a doctor in the out-of-hours GP cooperative. Referring to clinical knowledge or official guidelines on antibiotics or respiratory tract infections lowered expectations to what the caller could achieve by coming to the GP cooperative and strengthened the argument that self-management would be the best.

Sophia: It is okay. You say that they cannot come. Moreover, you have to give good advice and appear convincing. You cannot communicate; “I am a little insecure, but try to follow my advice anyway”. However, if you are very persuasive in how you give advice, supervise and communicate with the caller; my feeling is that it makes them feel more confident.

In all the groups, the informants mentioned safety netting as a third strategy in the negotiating process. The safety netting was described as presenting detailed information on signs of clinical worsening and how to act if it should occur, which created a sense of security for both nurse and caller. The informants felt that giving assurance of the possibility to call back at any time was often decisive for the callers’ acceptance of observing their condition at home. Several of the informants also pointed out the necessity of thorough documentation because this would prevent giving advice repeatedly and thereby ease the communication with future contacts.

Mia: The threshold is very low for calling back or coming if they are worried or if the clinical situation worsens. The door is open. They can come back, and I think most callers appreciate that. They feel a lot more reassured when they know they can come or call back. We have open doors day and night.

A fourth strategy was the use of laboratory tests without a clinical examination. Some informants considered it a last strategy if they were unable to reach consensus otherwise. The nurses invited the caller to come to the out-of-hours GP cooperative to test for streptococci or measure C-reactive protein (CRP). If the laboratory tests were negative, both the nurse and the caller would judge it as a decisive argument that a doctor’s appointment was unnecessary.

Olivia: But it does actually happen that you have someone on the phone whom you think can wait for the regular GP, but he strongly insists, and you offer or agree that he can come to measure his CRP. If there is nothing on the CRP, he will not get to see the doctor.

### Structural factors influencing the use of the out-of-hours service

In all the focus groups, the participants discussed structural factors influencing the use of the out-of-hours service.

A widespread opinion was that the fee-for-service payment of doctors made some doctors want more consultations in the out-of-hours GP cooperative. Several informants discussed how doctors might ask for more callers to be assigned for consultations to increase their income. The degree to which the doctors’ wishes influenced this aspect of the nurses’ professional practice varied among the informants. Some admitted that it was difficult not to lower the threshold for assigning callers for consultation, while others claimed that they were not influenced.

Olivier: They (the callers) are sick, after all, but they could very well have waited. However, it happens very often, at least with us, that the doctor wants to see patients because then they will receive more money.

In one of the out-of-hours GP cooperatives, the service plan had changed to grant the doctors a fixed salary on most duties, which had lowered the number of consultations. The impression was also that the doctors took more time for thorough clinical examinations, which in turn led to less inappropriate antibiotic prescribing.

According to Norwegian regulations, the out-of-hours GP cooperatives are obliged to respond to incoming calls within two minutes [11]. The informants discussed how this regulation increased the callers’ expectations of the out-of-hours cooperatives’ availability compared to their GPs’ offices. They also discussed how seeing a doctor after hours suited many people better than seeing a GP in daytime within normal opening hours. Several informants mentioned that strikingly many people got sicker around the time when they get home from work.

Eva: I feel that there must be convenience reasons when people call five minutes past four to say that they have had a sore throat and a fever since 10 am. I ask them, “have you called your GP?” And they say “No, it got a lot worse right now”.

The informants described epidemics, the population’s conception of low GP capacity and the fee-for-service plan in the out-of-hours service as non-controllable drivers of busy sessions. This busyness could lead to long phone queues. The informants discussed how they spent less time on counselling when there were long phone queues because they perceived it to be more time efficient to provide the callers with doctor’s appointments. The result was an increase in busyness inside the out-of-hours cooperative, which in turn lead to a lower capacity to answer phone calls from the public. It could be difficult to break this vicious cycle.

Eva: You do not have the time available that you should have spent on the phone, to maybe make them just stay home. There is this pressure the whole time. It is easier for us to just bring them in - and then chaos comes along.

### Callers’ characteristics influencing the use of the out-of-hours service

The informants described the characteristics of individuals or groups that considerably influenced the use of the out-of-hours service. A common theme in the groups was that the population had too little knowledge about the natural course of and self-care for respiratory tract infections. This inability of callers to distinguish between simple and serious respiratory tract infections created many worries and high expectancies of consultations in the out-of-hours service. The belief in a swift and painkilling effect of antibiotics, in addition to little knowledge of their side effects, were other reasons for the callers’ expectation of doctor’s appointments. The informants discussed how the same people paradoxically very often were concerned about the potential side effects of prescription-free painkillers. The callers’ previous experience with antibiotics against the same kind of symptoms was perceived as one of their strongest motivations to see a doctor. In such cases, it could be very difficult to persuade the caller to wait at home and observe their condition.

Lily: Often the caller says “the last time I needed antibiotics”. Then they think they had to have it to get well. They do not understand that they might have recovered anyhow. It does not help if I say so.

In all the groups, the informants talked about callers with limited Norwegian proficiency as a particularly challenging group. The language barrier made it very difficult to make proper assessments of their conditions over the telephone. Therefore, people from this group of callers usually received a doctor’s appointment.

Parents with sick children were described as another challenging group to communicate with. The informants discussed how some parents of young children wanted to place the responsibility of what might happen to their child with someone else. The conflict could often culminate if the nurse suggested that the parents could observe their child’s condition at home. Several of the informants described unpleasant experiences, where the caller had asked for a warranty that their child would not die the following night, in spite of modest symptoms. The situation could often turn into a discussion about responsibility. For the informants, it was difficult to keep their empathetic attitude towards the parents and their strong concern, and at the same time not let the parents shift the responsibility on to them.

Erin: I feel that some parents with children use it as an extra security: “Now I have at least told someone else, so now it is not my responsibility if something should happen. I have passed it on to health personnel, so now it is their responsibility if…”

A common theme in all the groups was how many callers could both exaggerate and underestimate the degree of urgency. The informants described this as challenging, especially because they felt that exaggeration was used strategically to get a doctor’s consultation.

Megan: I can hear that too. People have rather learnt what we ask. Because they say - almost before we say anything - all the things they know we will ask.

Erin: They build a clinical picture to force us to take them in.

Megan: Yes. They do.

Underestimation and understatements boosted the conception of phone assessments as demanding. Callers who reported minor symptoms but appeared at the out-of-hours GP cooperative gravely ill gave the informants an impression that there was an unpleasent level of chance in phone assessments. A seasoned nurse summed up her experience with people who underrated their own symptoms in this way:

Megan: Sometimes I think it is kind of by coincidence that we do the right things.

## Discussion

### Summary of main findings

The informants described a de facto gatekeeping role in the out-of-ours service. However, they considered themselves as service providers rather than gatekeepers. Negotiations and various strategies were used to maintain the balance between these two roles. The informants described callers’ characteristics and external factors interfering with the possibility to reach consensus.

### Gatekeeping and service providing – equilibrium on the tightrope

Health-care professionals and the public may have different views on which complaints warrant medical attention and care; thus, phone triage nurses experience the conflicting roles of being both carer and gatekeeper to limited health-care services as challenging [12]. Our informants recognised themselves primarily as service providers. The health-care system, however, also expects them to be gatekeepers due to the constantly higher demand for health care than the real supply. Sorting callers’ needs from demands, and to some extent protecting patients from antibiotic high prescribers, were situations where the informants seemed to accept the role as gatekeeper – however with ambivalence. Personal uncertainty was part of the aftermath when the nurses had performed gatekeeping, even when consensus had been reached. Serving the two diverging tasks as both carer and gatekeeper seemed to be a tightrope walk where nurses strived to find a balance.

Michael Lipsky’s theoretical ‘street level bureaucracy’ framework describes the dilemmas of the individual in public service [13]. Lipsky claims that the jobs of street level bureaucrats will not be performed according to the highest standards because of lack of time, information or other resources necessary to respond properly to the individual case. They will therefore use discretion in their work to balance the demands of the state and the demands of its citizens. The relationship between standardisation and discretion in triage nurses’ priority setting has been explored in depth elsewhere, concluding that discretion is an inevitable and necessary part of their assessments [14]. Our findings indicate that nurses use discretion more when they assess respiratory tract infections than when they assess cases of higher urgency, as the triage scales are of less use in the low urgency cases.

Phone triage nurses need to build a mental picture of callers and their needs to make good clinical assessments [12]. By nature, a phone call is a much more difficult setting with completely different communicational premises than that of a regular consultation. The regulation that demands 80% of incoming calls must be answered within 2 min, and the informants’ need to use discretion in the absence of relevant algorithms, are both factors making the phone assessment even more difficult.

Training has been shown to improve the quality of phone triage and increase the likelihood of managing phone calls definitively [15,16]. Our informants described that knowledge of relevant guidelines counterbalanced their reported difficulties of assessments and made it easier to reach consensus. Hence, specific training may strengthen phone triage nurses’ gatekeeping capacity.

Callers to the out-of-hours service with access to an emergency button to bypass the phone queue, use it with high accuracy, which indicates that they know when they are seriously sick [17]. Willingness to quarrel may be due to an underlying but non-expressed high degree of worry, which in turn can be a sign of a serious condition [18]. The phone nurses’ ability to listen to the callers’ worries, not their argument, is of vital importance. Hence, the informants’ consensus-seeking strategy for mastering the dilemma of gatekeeping and service providing is a constructive way of performing discretion, rather than an unwanted practice. One may argue that the tightrope equilibrium increases patient safety in the out-of-hours care.

The rapid CRP test is widely used in Norwegian primary health care. The CRP test is performed in 55% of consultations concerning respiratory illness in out-of-hours care [19], possibly giving rise to an understanding among the patients that the test is necessary to assess the severity of an infection. However, the guidelines on antibiotic prescribing in primary care recommend the test only as a supplement to clinical examination. The informants’ description of their use of a CRP test as part of a process of negotiations is comprehensible, but may be problematic. The results of a CRP test disconnected from a medical consultation has limited value and can be a false safety net for both the nurse and the caller [20].

### Organisational aspects of out-of-hours care

The demand for a two-minute response time is a measure designed to increase the availability of health care by ensuring swift contact for urgent cases. However, the regulation increased the informants’ perceived busyness. When external factors like epidemics entailed an increased number of incoming calls, they would often choose the less time-demanding solution of giving a doctor’s appointment instead of the more time-demanding choice of giving advice only [21]. They described this as a vicious cycle that increased the length of the queues both on the phone and in the clinic, which impaired patient safety and quality. However, callers want reassurance and support, and involvement in clinical decisions can increase their adherence to recommendations [22]. Hence, nurses in the out-of-hours service need sufficient time to make good clinical decisions to meet the needs of both the public and health-care services.

Organisational aspects of Norwegian primary health care in both staffing and financing areas have been discussed in recent years [23,24]. The organisation of local GP offices has been shown to influence how patients use out-of-hours GP cooperatives [25,26]. Our informants identified both the fee-for-service payment and the different organisation of local GP offices as factors that influenced how they perform triage and how they use discretion. These challenges show how neither the public nor the health-care personnel seem to perceive primary health care as one coherent service. This should be addressed in future discussions about GPs and leadership roles in primary care, leading to a closer collaboration between out-of-hours services and regular GPs.

### Caller’s characteristics

Callers’ characteristics influence triage nurses’ assessments [27]. Our informants described it as decisive for the outcome of their assessments, especially the problem of exaggeration and underrating of symptoms. Strengthening the communicative skills of phone triage nurses may increase the possibility to identify both ends of the symptom description scale. Such identification will increase the likelihood of recommending the right level of care, even for patients with poor symptom presentation. In light of the arguments for the consensus-seeking strategy, the habit of providing doctor’s appointment to low Norwegian proficiency callers is favourable and should be encouraged [28,29].

### Methodological considerations

The informants described how they assess callers with mild-to-moderate symptoms of respiratory tract infections, and their views and experiences on triaging and counselling these callers. There is always a risk that recalling and telling weakens the internal validity. Direct observations in an out-of-hours service could have provided a more realistic story of what actually happens when nurses perform triage. Our aim, however, was to explore the informants’ views and attitudes. We therefore judged focus groups as a feasible and pertinent method.

We chose a narrow study aim and a dense sample specificity. These factors, in addition to a strong and clear communication with the participants, indicate high information power in our material [30]. With 22 participants from different-sized out-of-hours cooperatives from various regions of Norway, we also think that our strategy of cross-case analysis is appropriate and strengthens information power. It would have been easier to perform all the interviews in local out-of-hours GP cooperatives only. We believe that our choice of including nurses from various parts of Norway has minimised the risk of internal culture in the groups and thereby strengthened external validity. Even with two local and two mixed groups, our impression is that there were no large differences between the groups. We did not include nurses from out-of-hours services in the two largest Norwegian cities because the populations of these cities tend to show up at out-of-hours clinics instead of calling in advance.

The authors’ background as GPs and head physicians of out-of-hours GP cooperatives has made it possible to penetrate and understand the informants’ stories and considerations. A nurse member of the research group might have opened more up for views disfavouring GPs. However, we perceived the conversations in the group interviews as open, and the informants spoke freely on conflicts between GPs and nurses.

All three authors have experienced out-of-hours work as busy and many of the consultations as medically unnecessary. In addition, we have found that busyness in the out-of-hours GP cooperatives leads to inappropriate antibiotic prescribing [4]. Our goal was to understand why nurses do not keep more callers out of the out-of-hours service, as it seemed to be a manageable task. This preconception was challenged when the informants revealed the complexity of their phone call assessments.

The findings of the current study form the basis of an educational intervention on respiratory tract infections for Norwegian phone triage nurses rolled out in out-of-hours GP cooperatives as part of an ongoing randomised controlled intervention.

## Implications

For phone triage nurses, low urgency cases may be as difficult to handle as high urgency cases. Triage algorithms in triage scales offer little support for mild respiratory tract infections. Nevertheless, these conditions are drivers of busyness. Hence, the triage algorithms should be improved to give better decision support also for these. Nurses in the out-of-hours service have a demanding and challenging task in the health-care system as de facto gatekeepers, and their consensus-seeking approach enhances patient safety in out-of-hours GP cooperatives. Such a strategy, however, demands both skills and time. Stakeholders must consider this when they make training plans for nurses and in planning the organisational and financial model, as well as the staffing, of out-of-hours GP cooperatives.

## Figures and Tables

**Table 1. Characteristics of the participants and the out-of-hours GP cooperatives.** 

Total number of participants	22
Median age	42 (24–66)
Male	5
Women	17
Median years of experience in out-of-hours service	7.5 (1–22)
Number of GP cooperatives represented	13
Small (< 50,000 inhabitants)	9
Large (> 50,000 inhabitants)	4
Median number of inhabitants of out-of-hours GP cooperatives	41,743 (22,205–76,649)
